# Graphene Layers Functionalized with A *Janus* Pyrrole-Based Compound in Natural Rubber Nanocomposites with Improved Ultimate and Fracture Properties

**DOI:** 10.3390/polym12040944

**Published:** 2020-04-18

**Authors:** Gea Prioglio, Silvia Agnelli, Lucia Conzatti, Winoj Balasooriya, Bernd Schrittesser, Maurizio Galimberti

**Affiliations:** 1 Department of Chemistry, Materials and Chemical Engineering Giulio Natta, Politecnico di Milano, Via Mancinelli 7, 20131 Milano (I), Italy; gea.prioglio@polimi.it; 2 Department of Mechanical and Industrial Engineering, University of Brescia, Via Branze 38, 25123 Brescia (I), Italy; silvia.agnelli@unibs.it; 3 Istituto di Scienze e Tecnologie Chimiche “Giulio Natta” (SCITEC), CNR, Via De Marini 6, 16149 Genova (I), Italy; lucia.conzatti@cnr.it; 4 Polymer Competence Center Leoben GmbH Roseggerstrasse 12, A-8700 Leoben (A), Austria; Winoj.Balasooriya@pccl.at (W.B.); Bernd.Schrittesser@pccl.at (B.S.)

**Keywords:** natural rubber, high surface area graphite, functionalization, serinol pyrrole, ultimate properties, fracture resistance

## Abstract

The ultimate properties and resistance to fracture of nanocomposites based on poly(1,4-*cis*-isoprene) from *Hevea Brasiliensis* (natural rubber, NR) and a high surface area nanosized graphite (HSAG) were improved by using HSAG functionalized with 2-(2,5-dimethyl-1H-pyrrol-1-yl)propane-1,3-diol (serinol pyrrole) (HSAG-SP). The functionalization reaction occurred through a domino process, by simply mixing HSAG and serinol pyrrole and heating at 180 °C. The polarity of HSAG-SP allowed its dispersion in NR latex and the isolation of NR/HSAG-SP masterbatches via coagulation. Nanocomposites, based either on pristine HSAG or on HSAG-SP, were prepared through traditional melt blending and cured with a sulphur-based system. The samples containing HSAG-SP revealed ultimate dispersion of the graphitic filler with smaller aggregates and higher amounts of few layers stacks and isolated layers, as revealed by transmission electron microscopy. With HSAG-SP, better stress and elongation at break and higher fracture resistance were obtained. Indeed, in the case of HSAG-SP-based composites, fracture occurred at larger deformation and with higher values of load and, at the highest filler content (24 phr), deviation of fracture propagation was observed. These results have been obtained with a moderate functionalization of the graphene layers (about 5%) and normal lab facilities. This work reveals a simple and scalable way to prepare tougher NR-based nanocomposites and indicates that the dispersion of a graphitic material in a rubber matrix can be improved without using an extra-amount of mechanical energy, just by modifying the chemical nature of the graphitic material through a sustainable process, avoiding the traditional complex approach, which implies oxidation to graphite oxide and subsequent partial reduction.

## 1. Introduction

Poly(1,4-*cis*-isoprene) from *Hevea Brasiliensis*, known as natural rubber (NR) [1,2], is the most important rubber, with a worldwide production of almost 14 million tons in 2018; it represents about 40% of the total rubber consumption [3,4]. Such a great success is based on the outstanding properties of NR: tack [5,6] and strength [7] in the uncured state and, upon vulcanizing, tensile strength [8,9] and resistance to fatigue and crack growth [10,11,12,13]. In particular, the latter properties are due to a peculiar property of NR, strain induced crystallization [14,15,16,17]. Indeed, NR is the selected rubber for demanding applications, for instance those in tire compounds (for cars, heavy trucks, and airplanes), anti-seismic base isolators, and anti-vibrating mounting pads. Moreover, NR is produced with a far lower energy input with respect to synthetic rubber: 15-16 MJ/kg versus a typical value of 100 MJ/kg [4]. It is also worth mentioning the efficient carbon sequestration performed by the *Hevea* tree: the photosynthetic rate of *Hevea* leaves is of about 11 μmol/m^2^·s versus a value of 5–13 μmol/m^2^·s in other trees [18]. Thus, the large amount of research performed on NR and NR-based composites can be easily understood.

In spite of its remarkable properties, NR is unable to meet the requirements of the above-mentioned applications. Better mechanical properties are required and achieved with the addition of reinforcing fillers [19,20]. Carbon black (CB) has been used for over a century [21]. After the discovery of fullerenes [22], an impressive amount of nanosized sp^2^ carbon allotropes has been prepared: single [23,24] or multi-walled [25,26] carbon nanotubes, graphene [27,28,29,30], and graphene-related materials [31,32,33,34]. Graphene is of particular interest because of its outstanding properties: an elastic modulus larger than 1 TPa, exceptional thermal and electrical conductivity [35,36].

Papers and reviews on rubber composites with graphene and related materials (GRM), mainly dedicated to isoprene rubber, are available in the scientific literature [37,38,39,40,41,42,43,44,45,46,47,48,49,50,51,52,53,54,55,56,57,58]. The basic objective of the reported researches is to obtain an ultimate distribution and dispersion of the graphene layers. Indeed, it is widely acknowledged that, by increasing filler distribution and dispersion, superior mechanical and ultimate properties and abrasion resistance can be achieved [59]. Moreover, the amount of filler can be reduced, thus preparing lighter materials. In the field of rubber composites, the filler dispersion can be improved by applying mechanical energy. However, such an approach can alter the structure of the rubber and even of the filler. Melt blending is used to promote the dispersion of nanographite, even with a very high surface area [39,44,50]. The solution mixing has been documented [58], but this approach is troublesome, particularly in view of an industrial scale up. The favored technology reported in the literature is the dispersion of graphene layers in latexes of NR [37,38,41,45,46,48,50,51,52,55] or of poly(styrene-*co*-butadiene) (SBR) [40], combined with the rational design of the surface chemistry of the filler [40]. In fact, to prepare latex dispersions, polar graphitic materials, in most cases graphene/graphite oxide (GO), are used [23,24,25,26,37,38,40,41,44,45,46,48]. GO, which is typically prepared by means of the Hummers and Offeman method [60,61,62,63] is then reduced, typically with chemical methods [64,65,66]. In such an oxidation-reduction method, the main disadvantage is the use of dangerous and even toxic chemicals (such as potassium permanganate, sulphuric acid, hydrazine), and harsh reaction conditions. Moreover, it is acknowledged that the bulk graphene structure is only partially restored: an appreciable amount of sp^3^ defects is present in the final product.

In this work, nanocomposites based on NR and graphene layers functionalized by means of a simple and sustainable method were prepared. The main objective of the research was to investigate the effect of an enhanced dispersion of the graphene layers on the dynamic-mechanical, tensile, and fracture properties of natural rubber nanocomposites. As the starting graphitic material, a high surface area graphite (HSAG) was selected, with a limited number of stacked layers (about 35) and a high order inside the basal plane, hence with a high shape anisotropy [66,67,68]. In order to achieve ultimate distribution and dispersion in the NR matrix, the nanocomposite was prepared via latex blending and, in order to obtain a higher compatibility with the latex, functionalization of HSAG was performed. A key objective of this work was to perform the functionalization with a sustainable and simple method, avoiding the above-mentioned oxidation-reduction cycle and harsh reaction conditions and preserving the structure of the graphene layers. Functionalization has been performed with a *Janus* molecule such as 2-(2,5-dimethyl-1*H*-pyrrol-1-yl)propane-1,3-diol (serinol pyrrole, SP), by simply mixing and heating HSAG and SP [69,70]. It has been shown [71] that a domino reaction occurs: a carbocatalyzed oxidation of the pyrrole compound occurs and then the pyrrole ring gives rise to a cycloaddition reaction with the graphitic substrate. The layers, whose bulk structure is substantially unaltered, are functionalized at the edges with OH groups. Nanocomposites prepared via latex blending have been compared with those obtained via melt blending. The dispersion of the graphitic filler was investigated with transmission electron microscopy (TEM). Dynamic-mechanical properties were studied through the application of stresses in the shear and axial modes, and tensile and fracture properties were also investigated. 

## 2. Materials and Methods 

### 2.1. Materials

High surface area graphite (HSAG) was Nano 27 from Asbury Graphite Mills, Inc. (Asbury, NJ, USA). According to the technical data sheet: carbon content is not lower than 99 mass%, surface area is 250 m^2^/g, and the chemical composition from elemental analysis (U.S. Standard Test Sieves) is carbon 99.82%, ash 0.18%, and moisture 0.97%. The number of stacked layers was estimated, as already reported [66,67,68], to be about 35.

The NR latex was a medium ammonia grade from Centex FA, with a 60 wt % solid content, pH (at 20 °C) = 9–11, a density of 0.95 g/cm^3^, and partial miscibility with water. 2,5-hexanedione (*M*_W_ = 114.12 g/mol) from Sigma-Aldrich (purity ≥97%, St. Louis, MO, USA), 2-aminopropane-1,3-diol (serinol, purity ≥98%) was kindly provided by Bracco (Milan (MI), Italy). Acetone was from Sigma-Aldrich (St. Louis, MO, USA), purity ≥97%. All the chemicals were used without further purification.

The following chemicals were used for the preparation of the elastomeric compounds: Zinc Oxide (from Zincol Ossidi, Bellusco (MI), Italy), stearic acid (from Sogis, Sospiro (CR), Italy), 6PPD (*N*-(1,3-dimethylbutyl)-*N′*-phenyl-p-phenylenediamine, from Crompton, Middlebury, CT, USA), sulphur (from Solfotecnica, Cotignola (RA), Italy), and TBBS (*N*-tert-butyl-2-benzothiazyl) sulfonamide, from Eastman (Sauget, IL, USA).

### 2.2. Synthesis of 2-(2,5-Dimethyl-1H-Pyrrol-1-yl)Propane-1,3-Diol or Serinol Pyrrole (SP)

A mixture of 2,5-hexanedione (HD, 12.15 g, 0.107 mol) and 2-aminopropane-1,3-diol (S, 9.96 g; 0.107 mol) was poured into a 100 mL round-bottomed flask equipped with a magnetic stirrer and a condenser (single glass tube). The mixture was then stirred (300 rpm) at 155 °C for 3 h. Then the condenser was removed, and the mixture was left stirring at 155 °C for 30 min. Afterwards, the reaction mixture was cooled down to room temperature. Finally, 15.51 g of pure, dark amber, viscous product was obtained. 1H NMR (CDCl_3_, 400 MHz); δ (ppm) = 2.27 (s, 6H); 3.99 (m, 4H); 4.42 (quintet, 1H); 5.79 (s, 2H). 13C NMR (DMSO-d6, 100 MHz); δ (ppm) = 127.7; 105.9; 71.6; 61.2; 13.9.

### 2.3. Functionalization of High Surface Area Graphite (HSAG) with Serinol Pyrrole (SP)

Two samples were prepared, with two different HSAG/SP mass ratios.

Sample 1. HSAG/SP mass ratio = 10/1. 10 g of HSAG and 50 mL of acetone were put in a 250 mL round-bottomed flask. A solution of 1 g of SP in 15 mL of acetone was then added. The suspension was sonicated for 10 min, using a 2 L ultrasonic bath 260 W (Sonica, Soltec Srl, Milan, Italy). The solvent was removed under reduced pressure using a rotary evaporator. The 250 mL round-bottomed flask was then equipped with a magnetic stirrer and a condenser, heated up to 180 °C in an oil bath, and left under stirring (300 rpm) for 3 h. One hundred mL of acetone were then added to the free-flowing powder and the suspension was stirred overnight at room temperature and then filtered on a Büchner funnel with a sintered glass disc. The free-flowing powder was recovered and dried in an oven at 95 °C for 4 h.

Sample 2. HSAG/SP mass ratio = 10/0.6. The same experimental procedure as above was used. Instead of 1 g of SP, 0.6 g was used.

### 2.4. Characterization of the HSAG-SP Adduct

Functionalization Yield was determined using Equation (1):

(1) $$\mathrm{Functionalization}\;\mathrm{Yield}\;\left(\%\right)=\frac{\mathrm{SP}\;\mathrm{mass}\;\%\;\mathrm{in}\;\left(\mathrm{HSAG}-\mathrm{SP}\;\mathrm{adduct}\right)\;\mathrm{after}\;\mathrm{acetone}\;\mathrm{wash}}{\mathrm{SP}\;\mathrm{mass}\;\%\;\mathrm{in}\;\left(\mathrm{HSAG}-\mathrm{SP}\;\mathrm{adduct}\right)\;\mathrm{before}\;\mathrm{acetone}\;\mathrm{wash}}$$

The mass percentages of Serinol Pyrrole (*SP*) in the HSAG-SP adduct (before and after acetone washing) were obtained from TGA analysis. This technique is frequently used to check the presence of organic compounds on the surface of carbonaceous fillers [72]. 

The TGA instrument used to perform thermogravimetric analyses was a Mettler TGA SDTA/851 (Mettler Toledo, Columbus, OH, USA). The standard method ISO9924-1 was followed. The method used for the analysis of adducts (5–10 mg) consists in a heating ramp (10 °C/min) from 30 up to 300 °C, followed by a 10 min isotherm at 300 °C, then another heating ramp (20 °C/min) up to 550 °C and another isotherm at 550 °C (15 min); this isotherm is followed by a final heating ramp (10 °C/min) up to 900 °C after which the temperature is kept constant until the end of the experiment. At 102.5 min from the beginning of the test, the gas in the chamber is switched from N_2_ to air. The whole experiment lasts 120 min.

### 2.5. Rubber Composites Preparation

Composites formulations, expressed in parts per hundred rubber (phr), are reported in Table 1.

Composites were prepared by using the same NR grade, in two steps. In the first step, either NR or NR/HSAG-SP were coagulated from the latex. In the second step, further ingredients were added through melt blending. The amount of HSAG and HSAG/SP was: 5, 15, and 24 phr. The intention was to use 25 phr of HSAG and HSAG/SP. In the HSAG/SP masterbatch coagulated from the latex, 24 phr were experimentally found (by means of TGA) and it was decided to use the same amount of HSAG in the corresponding composite.

#### 2.5.1. Coagulation of NR 

Dilution of NR latex. A typical procedure is as follows: 83.33 g of NR latex were poured in a 500-mL beaker, equipped with a magnetic stirrer; 100 mL of distilled water was added. The mixture was left under stirring (300 rpm) for 10 min at room temperature. 

Coagulation of NR from the latex. Rubber was then coagulated by adding 100 mL of a 1M sulphuric acid solution. The solid rubber was then squeezed, immersed in distilled water overnight, rinsed with water up to neutral pH, reduced to small pieces, and left to dry at room temperature. 

#### 2.5.2. Coagulation of NR/HSAG-SP 

The same procedure, reported as follows, was adopted for the preparation of three NR/HSAG-SP masterbatches (containing 5, 15, and 24 phr of HSAG-SP). 

*Preparation of HSAG-SP dispersion in water.* HSAG-SP was weighed and introduced in a 500-mL beaker, then distilled water was poured in the beaker, specifically 100 mL for each gram of adduct. The dispersion was sonicated in a 2 L ultrasonic bath with power of 260 W for 15 min to produce a homogeneous dispersion of the adduct in water. Then a magnetic stirrer was introduced in the beaker. 

*Dilution of NR latex*. A dispersion of 83.33 g of latex in 100 mL of distilled water was prepared as reported above.

*Coagulation of NR/HSAG-SP from the latex*. The diluted NR latex was added to the HSAG-SP dispersion in water. The mixture was left stirring (500 rpm) at room temperature for 1 h. Rubber was then precipitated by adding 100 mL of a 1M sulphuric acid solution. The precipitated rubber was washed and dried following the same procedure described above. 

#### 2.5.3. Melt Blending

*Composites with HSAG*. NR, coagulated from the latex, was fed into a 50 cc Brabender^®^ internal mixer and masticated for 1 min at 80 °C and 60 rpm, with 85% as the fill factor. HSAG was then added and mixing was performed for 3 min. Stearic acid, 6PPD, and zinc oxide were added, and mixing was carried out for a further 3 min. The composite was then discharged and fed again into the mixer kept at 45 °C. After 1 min mixing, TBBS and sulphur were added. After 3 min mixing, the composite was discharged.

*Composites with HSAG-SP*. The same procedure was followed, except for filler addition. The total mixing time was kept equivalent.

*NR composite.* The same procedure reported above was adopted, except for HSAG, which was not added. The total mixing time was kept constant.

### 2.6. Curing

The crosslinking reaction was performed and monitored with a rubber process analyzer (RPA, Alpha Technologies, Hudson, OH, USA) at 170 °C for 10 min. 5 g of crude rubber compound were introduced in the rheometer. Before the crosslinking step, a strain sweep was performed at low deformations (0.1–25% strain), then the sample was kept at 50 °C for ten minutes and subjected to another strain sweep at 50 °C. Subsequently vulcanization was performed during which the torque-time curve, the minimum achievable torque (*M*_L_), the maximum achievable torque (*M*_H_), the time needed to have a torque equal to *M*_L_ + 1 (*T*_S1_), and the time needed to reach 90% of the maximum torque (*T*_90_) were measured. Acquisitions were performed with an oscillation angle of 6.98% and a frequency of 1.7 Hz. 

### 2.7. Strain Sweep Tests

Shear dynamic-mechanical properties were measured using a rubber process analyser (RPA). A strain sweep was performed on the crude sample at low deformations (0.1–25% strain), then the sample was kept at 50 °C for ten minutes and subjected to another strain sweep at 50 °C before being vulcanized as described in the previous paragraph. After 20 min at 50 °C, shear dynamic-mechanical properties were measured, applying a 0.1–25% strain sweep at a frequency of 1 Hz. The measured properties were shear storage and loss moduli (*G*′, *G*″). 

### 2.8. Morphological Analysis

The dispersion of the carbon allotrope in the NR matrix was investigated by transmission electron microscopy (TEM, Carl Zeiss AG, Oberkochen, Germany) with an 80 kV Zeiss EM900 microscope. Ultrathin cryosections of the cured composites were prepared at −130 °C by using a Leica EM FCS cryo-ultramicrotome (Leica Microsystems, Wetzlar, Germany).

### 2.9. Dynamic Mechanical Analysis (DMA)

The experiments were conducted with a DMA 861/40N testing device (Mettler Toledo GmbH, Schwerzenbach, Switzerland) in tension mode. The parallel parts between the shoulders of the dumbbell specimens (S2) (thickness of ~2 mm and width of 4 mm) were utilized as the specimens with a clamping distance of 19.5 mm. In a first step, amplitude tests were conducted at room temperature within 1–100 μm identifying the linear viscoelastic range of the material grades. Based on the results, temperature sweep tests were carried out with a dynamic amplitude of 8 µm, a static amplitude of 103%, a frequency of 2 Hz, and a heating rate of 3 K/min within the temperature range of −80 to 50 °C using liquid nitrogen as a cooling agent. Out of the measurements, the storage modulus (*E*′), loss modulus (*E*″), and loss factor (tan*δ*) were plotted and compared in the investigated temperature range.

### 2.10. Quasi-Static Tensile Tests

The quasi-static tensile experiments were conducted based on DIN 53504 and the dumbbell-shaped test specimens (S2) were punched from the received ~2 mm thick plates. These specimens possess a width of 4 mm and were clamped at a distance of 43 mm. The tests were conducted with a Zwick universal testing machine (Zwick Roell Z001, Test expert, Ulm, Germany) utilizing a 1 kN load cell, at a constant crosshead speed of 200 mm/min according to the testing standard. The non-contact strain measurements based on the digital image correlation (DIC) technique were implemented using two cameras and the Mercury RT software (version 2.5, 2017) (Sobriety s.r.o., Kuřim, Czech Republic). Each material grade was characterized implementing five specimens to ensure reproducibility. Based on the results, stresses at 50 and 100% of strain (*σ*_50_ and *σ*_100_), as well as stress at break (*σ*_B_) and elongation at break (*ε*_B_) were determined in average values along with standard deviation.

### 2.11. Fracture Tests

**Figure 1:**
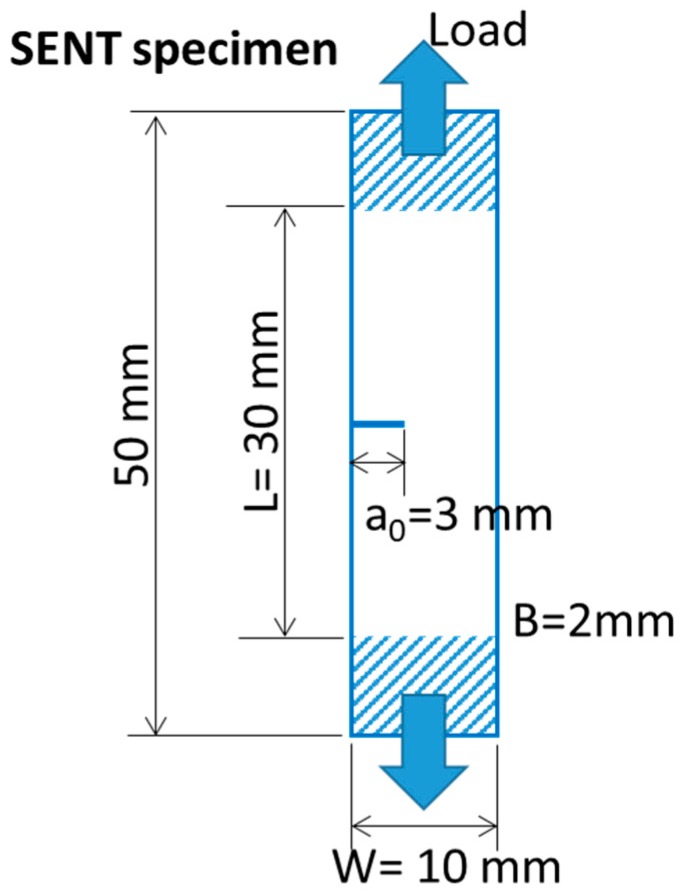
Sketch of the specimen used for fracture tests, with notch length (*a*_0_), thickness of the specimen (*B*), and width (*W*) and length (*L*_0_) of the specimen.

The experimental procedure, described in detail in [73], is a single specimen procedure developed to obtain the fracture resistance of rubber mode I condition, and is based on the J-integral parameter [74]. The procedure is summarized here: Single Edge Notched in Tension (SENT) specimens, shown in Figure 1, are used, with the following dimensions: width *W* = 10 mm, clamps distance *L* = 30 mm, thickness *B* = 2 mm, notch length *a*_0_ = 3 mm. SENT specimens were tested at 10 mm/min of crosshead rate. During loading, the load- crosshead displacement curve was monitored. J-integral values at the fracture initiation point, *J*_c_ (kJ/m^2^) values, were calculated by Equation (2):

(2) $$J_{c}=\frac{\eta \cdot U_{c}}{B\cdot \left(W-a_{0}\right)}$$

where *U*_c_ is the energy, the area under the stress-strain curve, up to the initiation point, and *η* is a geometry factor, previously calibrated for the dimensions used in this work (30 × 10 mm^2^) following the multispecimen procedure described in [73,75]. The *η* values are obtained from the following equation after measuring the actual *a*_0_ values from the fracture surfaces:

(3) $$\eta =-1.155\frac{a_{0}^{2}}{W}+1.8938\frac{a_{0}}{W}-0.0011$$

**Figure 2:**
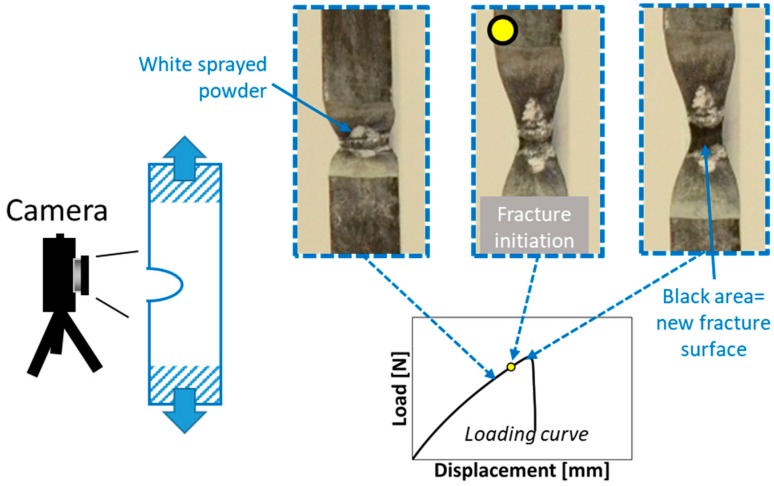
Experimental setup for fracture test: camera in front of the notch root, example of frames taken during a fracture test with the notch surfaces of a specimen. In particular, fracture initiation is indicated.

The point of fracture initiation was optically monitored by a camera placed in front of the notch taking an image every 2 s, as shown in Figure 2. The black notch surfaces were sputtered before testing by a white talc powder. In this way the new fracture surface can be easily distinguished as a black line at the notch root. Due to the progressive evolution of the fracture process, initiation is conventionally taken when the new fracture surface spans over all the specimen thickness and is 0.1 mm high (see ref. [73]). Five test repetitions were performed for each material.

## 3. Results and Discussion

### 3.1. Functionalization of HSAG

**Figure 3:**
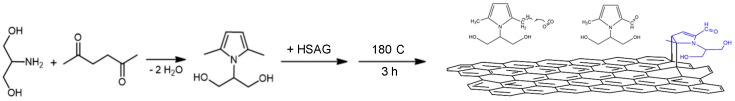
Scheme of the domino reaction for the functionalization of HSAG with serinol pyrrole.

The functionalization of HSAG was performed through the reaction with serinol pyrrole. As mentioned in the Introduction, the functionalization process is indeed simple and occurs through the domino reaction shown in Figure 3.

Details about the reaction are provided in the Materials and Methods section. In brief, serinol pyrrole is obtained by simply mixing and heating the primary amine (serinol) and the dicarbonyl compound and functionalization occurs by simply mixing the pyrrole compound and the graphitic substrate, then heating the mixture at 180 °C for 3 h. The domino functionalization process occurs via the following steps: adsorption of the pyrrole compound onto the graphitic material, carbocatalyzed oxidation of SP, and cycloaddition reaction with the graphene layers. In this work, SP was obtained by performing the reaction between the diketone and serinol in a flask, isolating the product. However, as already reported [76], the reactants can be mixed on top of the sp^2^ carbon allotrope. In this case, the first step of the domino process becomes the synthesis of the pyrrole compound. Such functionalization method avoids the above-mentioned harsh reaction conditions typically used for the traditional oxidation-reduction approach. In this work, two functionalization reactions were performed, with two HSAG/SP mass ratios: 10:1 and 10:0.6. The objective was to check the efficiency of the reaction by increasing the amount of the pyrrole compound. 

**Figure 4:**
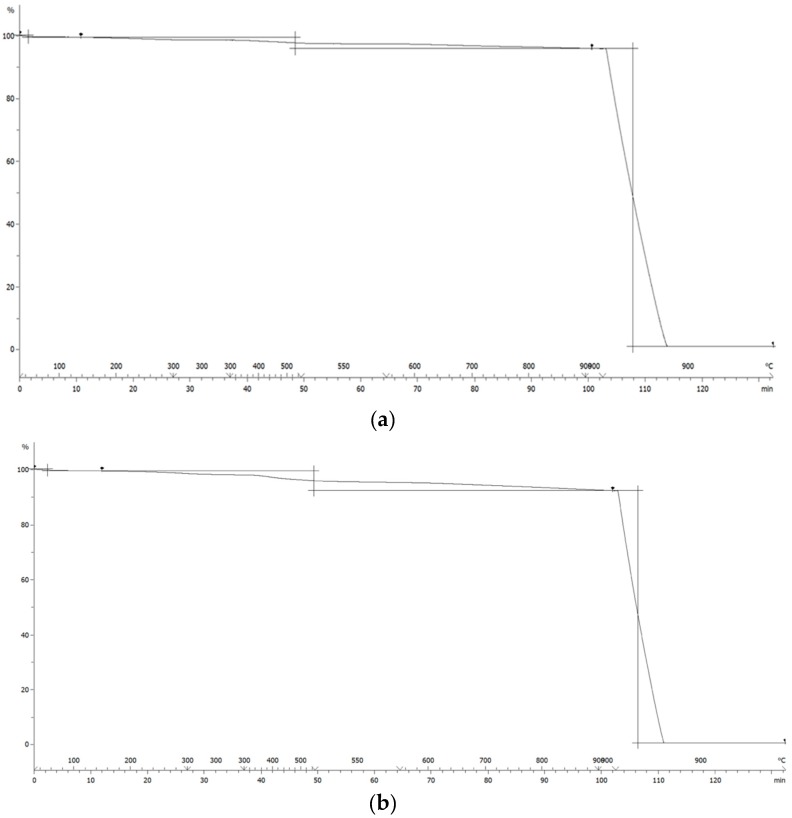
Thermogravimetric analysis of: (**a**) Pristine HSAG and, (**b**) HSAG-SP after acetone extraction (Sample 2 in Table 2 below).

The efficiency of the functionalization reaction was evaluated by means of thermogravimetric analysis (TGA), as described in Materials and Methods, on the HSAG/SP adduct isolated after the reaction and an exhaustive acetone extraction, curves are reported in Figure 4. The detected mass losses are in Table 2. 

Mass losses below 150 °C can be attributed to the removal of the adsorbed water. It is not revealed by washed pristine HSAG and increases with the amount of serinol pyrrole in the adduct. It is worth repeating (see Materials and Methods) that all the functionalization reactions were performed on washed HSAG samples. The mass loss at temperatures above 150 °C, for the washed sample of pristine HSAG can be attributed to alkenylic defects. In the case of HSAG/PyC adducts as well, mass loss is due to the presence of the organic modifier introduced with the functionalization reaction [69]. Such mass loss is indeed appreciable in the samples exhaustively extracted with acetone. Stable adducts were thus formed. The equation and procedure for estimating the functionalization yield was reported above in the Materials and Methods section. High functionalization yields were obtained by using the two levels of modifier, about 10% and 6% by mass: they were 88% and 87% for Sample 1 and Sample 2, respectively. For the preparation of the nanocomposites, Sample 2 (lower amount of SP) was used. This choice was motivated by two main objectives: to verify if such a low level of functionalization was enough to promote the dispersion of HSAG in the NR latex and then in the NR-based composite and then to reduce the interaction of the polar groups with the polymer chains. Indeed, the objective of this work was to investigate the effect of graphene layers on an NR-based composite. 

The infrared analysis of HSAG-SP adducts has been discussed elsewhere [70]. In the case of the samples prepared in this work, the typical spectral features of the pyrrole compound (PyC) derivative have been identified.

### 3.2. Preparation and Characterization of Rubber Composites

The same NR grade, coagulated from a latex, was used for the preparation of all the composites. Coagulation was performed of either pristine NR latex or NR/HSAG-SP dispersion. Melt blending was then carried out, adding the ingredients reported in Table 1 above. Three different amounts of HSAG or HSAG-SP were used: 5, 15, and 24 parts per hundred rubber (phr). The presence of the functional group in HSAG-SP was neglected and the same amount of HSAG and HSAG-SP were used. Hence, when discussing the results reported in the following paragraphs, it is important to bear in mind that in the case of composites based on HSAG-SP, graphitic content is slightly lower. 

#### 3.2.1. Curing

Sulphur-based crosslinking was performed with a typical recipe, based on sulphur and a sulphenamide. Data from rheometric tests, collected as described in Materials and Methods, are shown in Table 3.

The values of *M*_L_ and of *M*_H_ increase, as expected, with the amount of graphite. With pristine HSAG, higher *M*_L_ and *M*_H_ values were obtained for the largest content of the graphitic filler. The *M*_L_ values are usually correlated with the viscosity of the sample. It is also worth commenting that the M_H_ values are affected by the presence of a filler network when the considered filler content is above its percolation threshold, as the strain amplitude sweep is not large enough to completely disrupt the network. In a previous study [39], the percolation threshold of HSAG in a poly(isoprene) matrix was reported to occur at about 21 phr. The lower viscosity and lower *M*_H_ of the composite with HSAG-SP could be attributed to a better dispersion of the filler in the matrix. Differences, if any, are small amongst the values of *t*_s1_, *t*_90_ and (*M*_H_ − *M*_L_)/(*t*_90_ − *t*_s1_), which indicate, respectively, the induction and optimum vulcanization times and the vulcanization rate. It seems thus possible to comment that the functionalizing agent does not appreciably affect the curing kinetics. However, it can be observed that values of *t*_s1_ and *t*_90_ remain almost constant for the composites based on HSAG, whereas they decrease, though to a minor extent, in the case of composites based on HSAG-SP. In the field of elastomer composites, it is acknowledged that a sp^2^ carbon allotrope promotes faster sulphur-based vulcanization [77]. The decrease of *t*_s1_ and *t*_90_ for the HSAG-SP composites is thus in line with prior work and could indicate a better interfacial area between the filler and the composite ingredients, and hence a better filler dispersion.

#### 3.2.2. Strain Sweep Tests 

Strain sweep experiments were performed, using a rubber process analyzer (RPA), as described in the Materials and Methods section. Storage (*G*′) and loss (*G*″) moduli and Tan Delta_max_ (*G*″/*G*′) were measured. The obtained results are reported in Table 4. Low levels of moduli were obtained, as expected, in consideration of the low amount of the graphitic filler. It should be also considered that the nanographite is made of graphene nanoplatelets, disposed parallel to the rotors of the RPA. Composites with lamellar nanographite have been reported to have mechanical anisotropy, revealing an orthotropic and transversally isotropic response: modulus values were observed to be very similar in all directions in the sheet plane and much larger (almost double) in the orthogonal direction [66].

Values of Δ*G*′ and Tan Delta_max_ increase with the HSAG content, as commonly observed in filled rubbers. The Δ*G*′(*G*′_0.1%_ − *G*′_25%_) value is taken as an indicator of the so-called Payne Effect, which expresses the non-linearity of the viscoelastic modulus due to the disruption of the filler network formed by the interaction of the filler particles, either directly or mediated by polymer layers [78,79]. Hence, the behavior at low strain of the nanocomposites, based on either HSAG or HSAG-SP, is similar at the same filler content.

#### 3.2.3. Dynamic-Mechanical Tests

**Figure 5:**
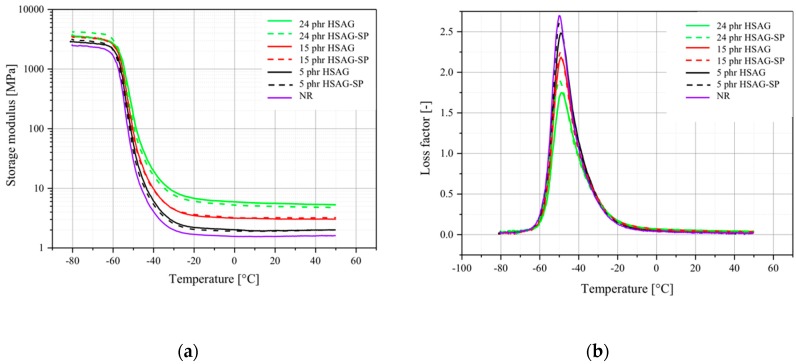
Dynamic mechanical analysis of vulcanized NR-based composites containing either HSAG or HSAG-SP (**a**) Storage modulus deviation versus temperature; (**b**) Loss factor deviation versus temperature.

The mechanical properties of rapidly varying stress conditions over a broad range of temperatures of the material grades were studied using the DMA method. The storage modulus (*E*′) and loss factor along the temperature range are shown in Figure 5a,b, respectively. The storage modulus reveals the elastic component of material behavior, which is related to the stiffness of the material. The peak of the loss factor corresponds to the glass transition temperature (*T*_g_), and the peak intensity, as well as the width, revealing the damping properties of the material [80,81].

*E*′ is affected by the presence of nanographite, independent of the functionalization: the storage modulus increases with the nanofiller amount, to a larger extent above the glass transition temperature. In the case of 5 phr- and 15 phr-filled nanocomposites, the functionalization with serinol pyrrole only slightly influenced the E′ values, whereas a lower E′ was obtained at 24 phr with HSAG-SP, even though the difference is still pretty low. 

The *T*_g_ appears to be substantially unaffected by the nanofiller type and content, except for the nanocomposite containing 24 phr of filler: in the presence of HSAG-SP, the *T*_g_ value was about 1.5 °C lower than that obtained with HSAG. The intensity of the loss factor peak clearly depends on nanographite type and amount. Such intensity decreased by increasing the HSAG amount. As to the filler type, larger damping was obtained with HSAG-SP, at all the filler contents. 

**Figure 6:**
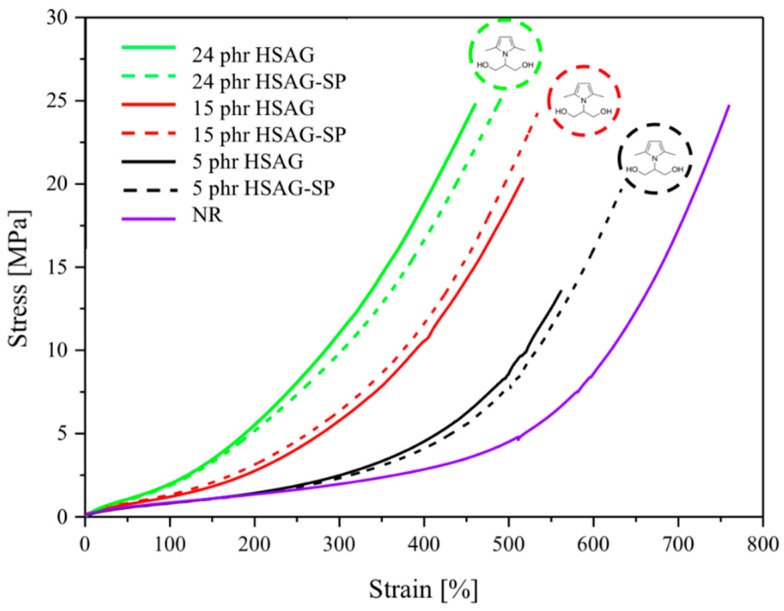
Stress-strain curves of vulcanized NR-based composites containing either HSAG or HSAG-SP. The number of each composite is close to the corresponding curve. Moreover, the chemical structure of serinol pyrrole is shown close to the curves of composites based on HSAG-SP.

These findings are in line with what is reported in the literature for composites based on graphene related materials (GRM) and NR. Enhancement of *E*′ with GRM content, to a different extent, was found by using NR latexes; and thermally reduced GO, up to 4 phr (in prevulcanized natural rubber) [45], chemically reduced GO, up to 2 phr [43] or 5 phr [46], GO, up to 0.5 phr [54]. Particular enhancement of *E*′, of two orders of magnitude, was found with GO up to 5% in the composite. In this case, it was commented that GO acted as a physical crosslinker [51]. Indeed, the interpretation for the *E*′ enhancement is based on the hydrodynamic effect promoted by rigid inextensible filler particles and on their interaction with the polymer chains. The larger *E*′ increase, with respect to NR, observed in this work, in comparison with those commented above for composites based on reduced graphene oxides, has to be attributed to the larger content of HSAG (up to 24 phr). To explain the larger *E*′ observed in Figure 6 for the composite with 24 phr HSAG, a lower mobility could be hypothesized for the polymer chains trapped in the network of HSAG aggregates. In fact, the HSAG percolation threshold in a poly(isoprene) matrix was reported to occur at about 21 phr [39]. Less aggregates and more exfoliated layers are present in HSAG-SP based composites, as is shown by TEM pictures (discussed in the text below). It is also worth adding that HSAG-SP was reported to exfoliate in a water suspension [68]. Further explanation for the larger *E*′ of HSAG-based composites could be based on the consideration that graphene layers stacked in a crystalline aggregate are estimated to have larger volume than exfoliated layers. Hence, HSAG aggregates could be expected to give more mechanical reinforcement than exfoliated HSAG. 

In almost all the mentioned papers [43,51,54], *T*_g_ was found to remain at the same temperature. A shift to slightly higher temperature was found in the presence of a surfactant in Ref. [45], which was commented to improve the interaction between the filler and the polymer chains. The slight reduction of *T*_g_ commented above for the HSAG-SP based composite could be interpreted by the larger mobility of the polymer chains, which are in the presence of a larger amount of exfoliated graphene layers. 

The reduction of tan delta peak value is a common feature in all the examined papers and is explained by the good interaction between the filler and the polymer chains. According to the literature [80,81], the source of damping is the reversible interaction of polymer chains with the filler surface. A more stable polymer-filler interaction is expected to reduce the loss factor intensity. Hence, it could be commented that a better HSAG-SP dispersion and exfoliation could favor the chain segment motion, thus reducing *T*_g_. At the same time, the larger extent of reversible interactions between the HSAG-SP layers and the polymer chains leads to larger damping.

The above findings present the peculiar behaviour of HSAG-SP: functionalization with the polar pyrrole compound allows to improve the dispersion of the filler without establishing a strong interaction with the NR chains; in the case of GO, it leads to higher *T*_g_ values. 

#### 3.2.4. Quasi-Static Tensile Tests

Quasi-static tensile measurements were performed on vulcanized composites, based on HSAG and HSAG-SP. A gum stock, with vulcanized NR and without any filler, was tested for comparison. Data from the measurements, with standard deviation, are given in Table 5 and representative stress-strain curves in Figure 6. Upon adding nanographite (with or without SP) to NR, higher stresses at every strain were obtained. HSAG and HSAG-SP based composites show comparable values of stress for a given value of deformation. As far as the ultimate properties are concerned, the strain at break decreased for all the composites loaded with the graphitic filler. The stress at break of the composites with 15 and 24 phr of graphite has substantially the same value as the NR gum stock, whereas it is lower for the composite with 5 phr, particularly with HSAG as the filler. As a matter of fact, at all the filler loadings, HSAG-SP led to higher values of elongation at break and stress at break, with respect to pristine HSAG. 

As far as the strain at break is concerned, the results in Figure 5 and Table 5 are in line with those reported in the literature, although the level of filler content here explored is much larger. In most literature works based on composites from NR latexes, the strain at break was found to decrease, to a different extent, with the content of GRM, by using thermally [45] or chemically [46] reduced graphene oxide, mechanically exfoliated graphite [53] or GO [48,51]. In these works, the maximum amount of the graphitic material was 4–5 phr (or %). When the GRM content was lower than 1%, the strain at break was substantially unaltered [51,54,55]. An increase of *ε*_B_ was also reported, however with graphene nanosheets prepared through the oxidation of graphite worms and dispersed with the help of ultrafine carbon black and silane as coupling agent and adopting 4 phr as the maximum content of the filler [57]. In the case of composites based on synthetic isoprene rubber and the same HSAG used in the present work and prepared via melt blending, a consistent reduction of *ε*_B_ was observed with a filler content up to 60 phr [44]. 

The examination of literature stress at break data does not allow a straightforward summary and this hampers the comparison with the results reported in this manuscript. With reduced graphene oxide, the value of stress at break increases and then decreases, moving from 1 to 4 phr in ref [45]; an optimum level of 0.5 phr is reported in [46], where the strain induced crystallization of NR is supposed to play a main role. By using GO, with a filler concentration of 0.5%, an exceptional increase was found for the tensile strength, which decreased then to a value not far from that of NR, at 5% as GO content. The effect of GO on the tensile strength was the increase for a content of 1% and then a decrease to a value lower than that of NR at 5% [51]. The hybrid filler with a graphene layer, ultrafine carbon black, and the silane coupling agent led to a simultaneous and consistent increase of elongation and stress at break [57]. In the work with HSAG in synthetic poly(isoprene) a consistent decrease of both elongation and stress at break were found [44]. The present work shows that the stress at break can have substantially the same value of the NR gum stock even at 24 phr as filler content. The better ultimate properties obtained with HSAG-SP could be attributed to better filer dispersion. This could explain in particular the difference observed at 5 phr as the filler content. It could be hypothesized that, at this filler content, HSAG does not achieve a good dispersion in a composite prepared via melt blending, because of the low shear stress experienced during the mixing. The lower shear stress found also with HSAG-SP appears to be in line with some of the literature reports. It could be speculated that the increase of the tensile strength at larger filler contents could be mainly due to the increased interactions between the filler and the polymer chains. However, further experiments are needed, not to stretch too far inferences not supported by experimental evidences.

#### 3.2.5. Mooney Rivlin Plot

**Figure 7:**
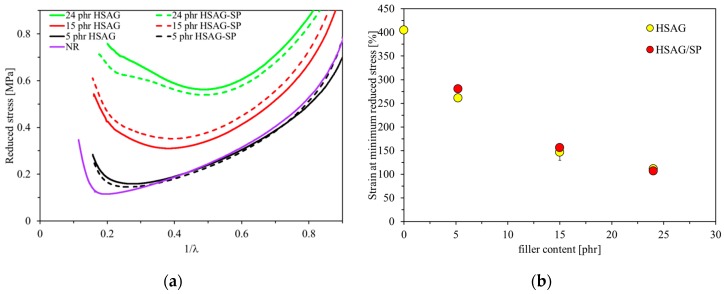
(**a**) Mooney–Rivlin plots of reduced stress versus reciprocal extension ratio of unfilled NR and nanocomposites based on either HSAG or HSAG-SP. Only representative curves are reported. (**b**) Strain at the minimum reduced stress. This parameter is conventionally taken as indication of the stress upturn point.

The stress–strain curves were converted into the Mooney–Rivlin plots, shown in Figure 7, where the reduced stress given by Equation (4)

σ* (σ* = *σ*/(*α^2^* − *α*^−1^)) (4)

was plotted versus the reciprocal of the extension ratio *α*.

The Mooney Rivlin plot reveals the stress upturn, which is considered to be due either to the entanglements which behave as effective crosslinking points at higher elongations. [82] or to the finite extensibility of the network chain segments [83,84] or to the strain induced crystallization [82,83,84,85,86,87,88]. 

Curves in Figure 7 refer to the NR gum stock and the composites based on HSAG and HSAG-SP, with different filler contents. In the graph in Figure 7a, a remarkable decrease in stress at low strain levels can be observed, attributed to the Payne effect in the case of filled rubbers. Furthermore, NR and composites with low amount of filler (5 phr) show a nearly linear region, followed by a stress upturn. The higher the filler content, the less definite is the central linear region of the plot, which turns into a smooth curvature. It appears that the upturn occurs at a lower extension ratio for the HSAG-based nanocomposites: the larger the amount of filler, the lower is the extension ratio. At 5 phr of filler content, the upturn occurs at an appreciably lower strain in the case of HSAG-SP. 

In the literature, the Mooney-Rivlin plot has been elaborated upon for NR/GRM composites from latex blending, containing a low amount of GRM. With 1 phr of either GO or chemically reduced GO [89], the upturn occurred at a lower strain than in the case of NR, in particular for the reduced GO. Decrease of stress upturn was found by increasing the amount of reduced GO from 1 to 2 phr [43]. Abrupt upturn was observed with 0.5 phr of reduced GO [46]. These findings were attributed to the strain-induced crystallization, a phenomenon that was documented to be favored by such a level of GRM content [90]. However, when the content of reduced GO was increased up to 5 phr [46], a gentle instead of an abrupt upturn was observed and attributed mainly to limited extensibility of the network chains. In this study, this latter interpretation could be adopted to explain the results of Figure 7. The difference observed between HSAG and HSAG-SP based composites, at 5 phr as the filler content, could be explained by the larger mobility of polymer chains in the latter.

#### 3.2.6. Fracture Tests

In the rubber field, natural rubber is the material of choice to prevent crack propagation. The fracture behaviour of the NR–HSAG and NR–HSAG-SP composites was studied by applying a fracture mechanics approach based on J-testing methodology [73]. Fracture tests allow to evaluate the resistance of rubber in the presence of a defect. Due to the innovative character of the materials and their limited availability, fracture resistance was characterized by the single specimen method based on the J integral parameter, described in Ref. [73] and in Materials and Methods, on quite small specimens. The J integral parameter describes the energy required to produce a unitary fracture surface. Due to the viscoelastic nature of rubber and the size of specimens—smaller than usual—the results are also influenced by energy dissipation in the specimen bulk. However, since all the materials were tested with the same specimen dimensions, the results can be useful to rank the materials on the basis of the energy required to initiate fracture.

**Figure 8:**
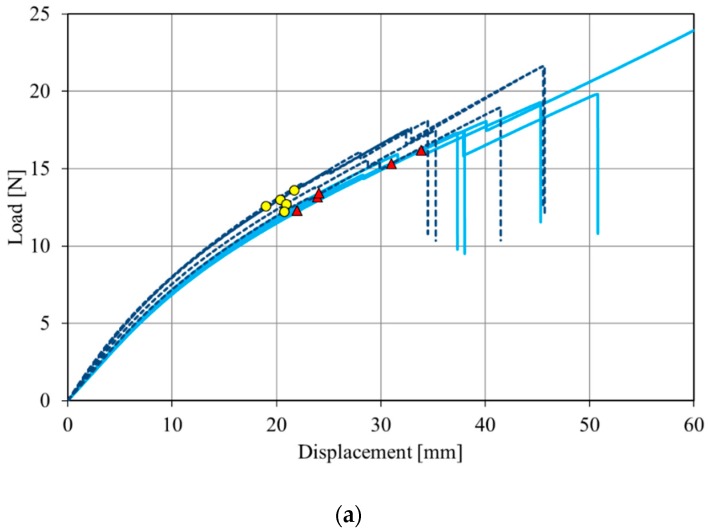

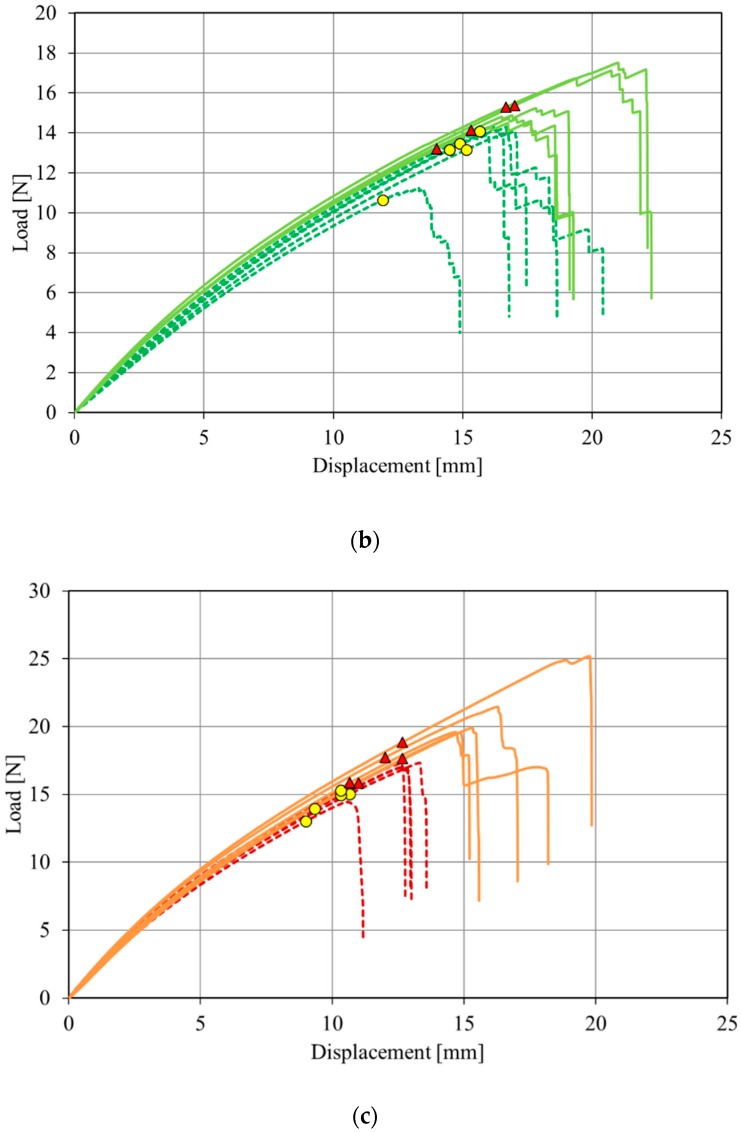
Fracture curves for NR-based composites containing 5 (**a**), 15 (**b**), and 24 phr (**c**) graphitic nanofiller. Lighter continuous lines and darker dashed lines refer to composites with HSAG-SP and HSAG, respectively. Dots and triangles on the curves indicate the points of fracture initiation for HSAG and HSAG-SP, respectively.

Loading curves obtained from fracture tests are reported in Figure 8: Figure 8a–c for composites with filler content, either HSAG (darker curves) or HSAG-SP (lighter curves), equal to 5, 15, and 24 phr, respectively. The yellow dots and red triangles on the curves indicate the points of fracture initiation.

The elongation before fracture decreases by increasing the amount of the graphitic material for all the composites—the material becomes stiffer. It is worth pointing out that the functionalization of HSAG with SP appears to improve the fracture behaviour: curves with and without SP at the same filler amount are similar, but fracture occurs at larger deformation and with higher values of load in the case of HSAG-SP based composites. This causes slightly higher values of fracture resistance, *J*_c_. Average fracture toughness (*J*_c_) values obtained from Equation (2) are reported with the corresponding deviations in Table 6.

Composites containing HSAG-SP are tougher; they show higher values of stored energy before fracture initiation with respect to composites containing pristine HSAG, and this difference is significant at 24 phr. It is acknowledged that in tensile tests, undispersed filler particles promote specimen failure. Hence, these experimental findings seem to suggest a better dispersion of HSAG-SP. The difference between HSAG and HSAG-SP based composites appears more relevant for 24 phr, as the filler content. As already reported, the percolation threshold of HSAG in a poly(isoprene) matrix occurs at about 21 phr [39]. Hence, the beneficial effect of a better filler dispersion seems to be more evident above the filler percolation. Similar conclusions were achieved in a recent study [54], which compared the fracture properties of NR/GO nanocomposites via melt and latex compounding. The same fracture method applied in the present work was used, but with different specimen dimensions and at very low filler content (< 0.5 phr). In spite of these differences, an improvement in fracture properties with latex blending due to better nanofiller dispersion was observed. It is reasonable that a polar filler such as GO benefit from the blending in rubber latex. This was also the hypothesis of the origin of this work, inspired also by the chance of using a polar graphite functionalized in a simpler and more sustainable way, with respect to GO.

Values of pure NR are not reported, although the experiments were performed under the same experimental conditions. As a matter of fact, NR specimens were elongated up to about 700% at complete break during fracture tests. At such a high deformation, the specimen completely lost its shape and stress state completely changed. This behaviour can be attributed to the extremely high deformability of NR combined with a high fracture toughness, and to the small size of the specimens, which does not provide enough constraint during the test. Therefore, although a value of fracture toughness equal to 18.1 ± 1.2 kJ/m^2^ was calculated for NR, this figure was considered to be not reliable and thus not comparable with the other results. It is, however, clear that NR has a higher fracture toughness compared to the other composites, most likely as a consequence of its strain-induced crystallization.

**Figure 9:**
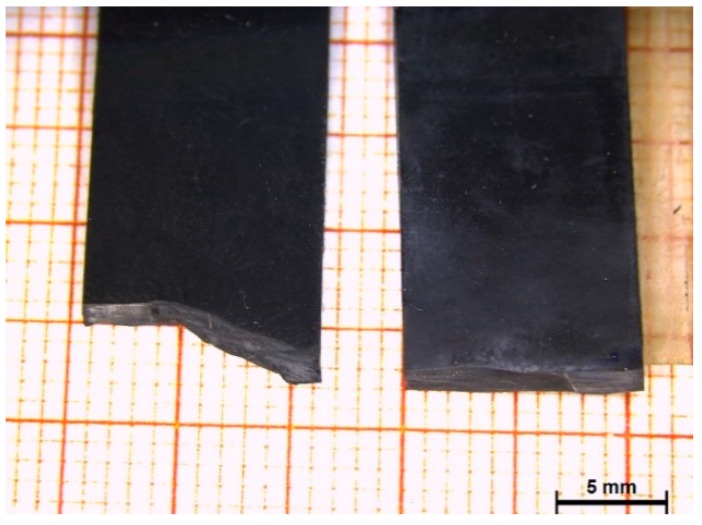
Side view of fractured samples containing 24 phr of HSAG-SP (**on the left**) and HSAG (**on the right**).

The morphology of the fracture surface was investigated. For neat NR and composites with 5 and 15 phr of both fillers, and composite with 24 phr of HSAG, the fracture propagated straight and perpendicular to the applied load. Only the composite with 24 phr of HSAG-SP showed a different behaviour. Pictures of fractured samples of composites with 24 phr of filler are shown in Figure 8 (side view). The straight fracture propagation visible in the NR/HSAG composite indicates that the crack follows the shortest possible path (Figure 9 right), whereas the change of direction is evident in the specimen with HSAG-SP (Figure 9 left). 

Fracture propagation deviates in HSAG-SP composites with 24 phr of filler, as if it had encountered obstacles that force the change of direction. This makes the material tougher: a higher amount of energy is required to break the material. To account for such a behaviour, it could be useful to remember that NR gives rise to strain-induced crystallization (SIC). HSAG-SP based samples achieve greater elongation; this could favour the crystallization. Moreover, the dispersion at the nano level of two-dimensional reinforcing fillers has been shown to favor SIC [46,48]. The crystallites could be responsible for the modification of the direction of propagation of the fracture found at 24 phr of filler content. 

#### 3.2.7. Structure of the Composite; TEM Analysis

**Figure 10:**
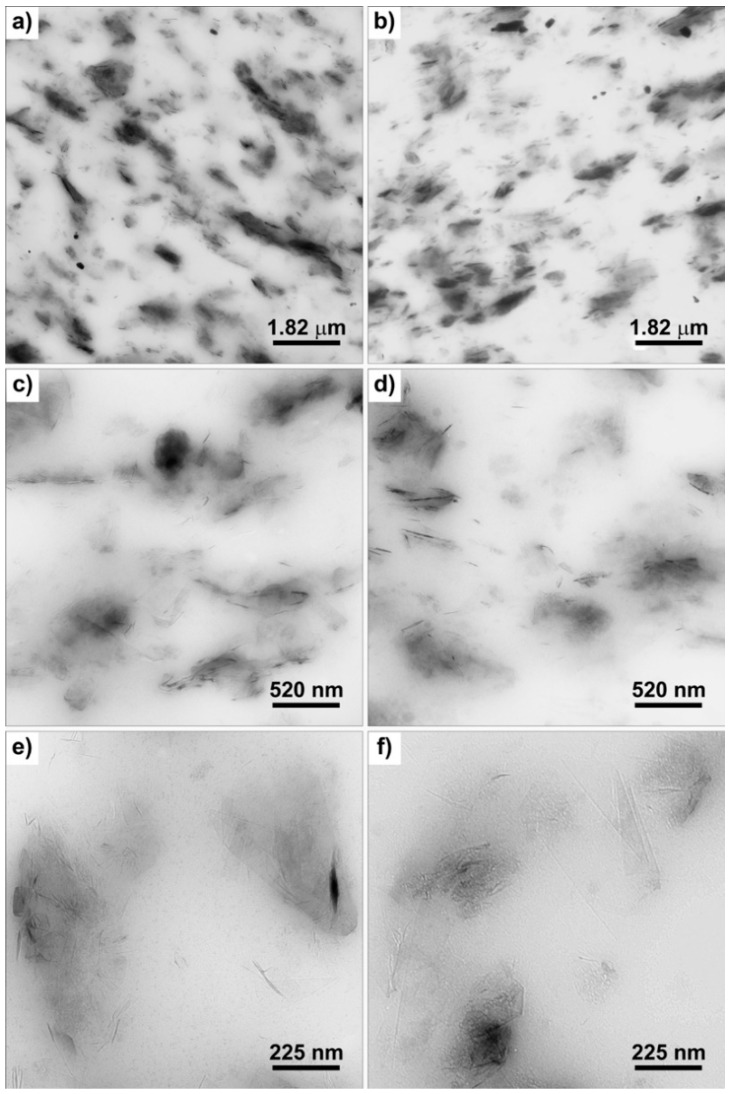
TEM micrograph taken at different magnifications of the samples containing 24 phr of HSAG (**a**,**c**,**e**) and HSAG-SP (**b**,**d**,**f**).

Experimental findings from dynamic-mechanical, tensile, and fracture tests suggest that, thanks to functionalization with SP, a good dispersion of the graphitic filler was achieved. To confirm such hypothesis, the structure of cured composites based on HSAG or HSAG-SP was investigated by TEM analysis at different magnifications. Representative micrographs of the composites containing 24 phr of nanofiller are shown in Figure 10.

A relatively homogenous distribution of sub-micrometric aggregates was observed at low magnifications (Figure 10a) for both the samples, suggesting the presence of a well-structured nanofiller network. The morphological analysis carried out at different magnifications indicates a similar nanofiller dispersion in the two samples; however, in the sample containing HSAG-SP (Figure 10b,d,f) smaller aggregates and higher amounts of few layers stacks and isolated layers can be observed within the elastomer matrix.

## 4. Conclusions

This work reports a simple and sustainable way of improving the ultimate and fracture properties of a nanocomposite based on natural rubber and a graphitic material. The nanocomposite is characterized by the ultimate dispersion of a nanosized high surface area graphite, achieved by modifying the chemical nature of the carbon allotrope, thus without using an extra amount of mechanical energy. The polarity of HSAG was increased, thanks to functionalization with serinol pyrrole; a masterbatch of HSAG-SP in NR was prepared by coagulating the HSAG-SP dispersion in NR latex. The functionalization with the pyrrole compound, with respect to the traditional approach based on GO, has the following main features: it does not require a reduction step (either thermal or chemical) and the use of hazardous chemicals and harsh reaction conditions, and it leaves the bulk structure of the graphitic substrate substantially unaltered. The polar HSAG-SP does not act as GO, as a crosslinking agent for the polymer chains, but promotes the ultimate dispersion of the graphene layers. Composites, with either pristine HSAG or HSAG-SP, were then prepared with melt blending, using traditional equipment and energy. Good dispersion of the graphene layers appears to be responsible for better ultimate and fracture properties of the sulphur-crosslinked HSAG-SP composites. These results were obtained by using HSAG with a low amount of SP and by preparing the dispersion of HSAG-SP in water and then in NR latex by means of magnetic stirring. Recent studies [34,91] have demonstrated that the shear rate of order of magnitude of 10^4^ s^−1^ can lead to extensive exfoliation of the graphitic aggregates and that the affinity of graphite for the solvent (obtained through functionalization) is highly beneficial. The perspective of the present research proceeds by obtaining a larger exfoliation of graphitic adducts with SP by using appropriate technology, also in view of a scale up, which appears feasible. Through tailor-made functionalization of the graphitic material, with the appropriate pyrrole compound, composites based on different elastomers will be prepared, with evenly dispersed graphene layers and hybrid filler systems, aimed at reproducing the improvements of the properties shown in the present work.

## Figures and Tables

**Table 1:** Recipes of NR-based composites with HSAG or HSAG-SP as reinforcing fillers ^a,b^.

Composite	0	1	2	3	4	5	6
NR	100	100	100	100	100	100	100
HSAG	/	5	15	24	/	/	/
HSAG-SP	/	/	/	/	5 ^c^	15 ^c^	24 ^c^

^a^ Amount of ingredients in phr ^b^ Other ingredients: Stearic Acid 2, Zinc oxide 4, 6PPD 2, TBBS 1.7, sulphur 1.2 ^c^ Theoretical values. For the experimental values, see below in the text.

**Table 2:** Mass losses for HSAG and HSAG-SP adduct, as detected from TGA analysis.

Sample		Mass Loss (%)			
		T < 150 °C	150 °C < T < 400 °C	400 °C < T < 700 °C	T > 700 °C
HSAG	(a)	1.3	1.7	4.6	92.4
	(c)	0.0	0.1	0.4	99.5
HSAG-SP Sample 1	(b)	0.9	6	9.4	83.6
	(c)	1.0	5	8.5	85.5
HSAG-SP Sample 2	(b)	0.5	2.1	4.1	93
	(c)	0.6	1.4	4	94

(a) Pristine (b) after the functionalization reaction (c) after washing with acetone (see Materials and Methods section).

**Table 3:** Curing data of NR–HSAG and NR–HSAG-SP compounds.

Composite n.	1	2	3	4	5	6
**Filler Type**	HSAG	HSAG	HSAG	HSAG-SP	HSAG-SP	HSAG-SP
**Filler Amount**	5 phr	15 phr	24 phr	5phr	15 phr	24 phr
***M*_L_ (dNm)**	0.3	0.8	1.3	0.6	0.9	1
***M*_H_ (dNm)**	6.1	8.4	10.2	6.4	8.2	9.1
***M*_H_ − *M*_L_ (dNm)**	5.8	7.6	8.9	5.8	7.3	8
***t*_90_ (min)**	3.9	4.0	3.8	4.3	4.2	3.8
***t*_S1_ (dNm)**	2.5	2.5	2.3	2.7	2.5	2.3
**(*M*_H_ − *M*_L_)/(*t*_90_ − *t*_s1_)**	4.1	5.1	5.9	3.6	4.3	5.3

**Table 4:** Storage modulus *G*′ at minimum (0.1%) shear strain amplitude, Δ*G*′(*G*′_0.1%_ − *G*′_25%_), maximum loss modulus *G*″, and maximum Tan Delta_max_ (*G*″/*G*′) from strain sweep tests.

Composite n.	1	2	3	4	5	6
**Filler Type**	HSAG	HSAG	HSAG	HSAG-SP	HSAG-SP	HSAG-SP
**Filler Amount (phr)**	5	15	24	5	15	24
***G*′_0.1%_**	0.3	0.5	0.9	0.4	0.6	0.7
**Δ*G*′ = (*G*′_0.1%_ − *G*′_25%_)**	~0	0.1	0.3	~0	0.1	0.2
***G*″_max_**	0.02	0.03	0.06	0.02	0.03	0.05
**Tan Delta_max_**	0.06	0.06	0.08	0.05	0.06	0.08

**Table 5:** Stress at 50% (*σ*_50_) and 100% (*σ*_100_) strain, stress at break, and strain at break of NR/HSAG and NR/HSAG-SP vulcanized samples.

Composite n.	0	1	2	3	4	5	6
**Filler Type**	NR	HSAG	HSAG	HSAG	HSAG-SP	HSAG-SP	HSAG-SP
**Filler Amount (phr)**	0	5	15	24	5	15	24
*σ*_50_ (Mpa)	0.57 ± 0.02	0.6 ± 0.01	0.7 ± 0.10	1.1 ± 0.06	0.5 ± 0.02	0.8 ± 0.03	1.0 ± 0.02
*σ*_100_ (Mpa)	0.8 ± 0.02	0.8 ± 0.01	1.2 ± 0.06	1.9 ± 0.10	0.8 ± 0.01	1.3 ± 0.03	1.9 ± 0.04
*σ*_300_ (Mpa)	1.93 ± 0.08	2.53 ± 0.04	6.04 ± 0.31	10.5 ± 0.6	2.31 ± 0.02	6.19 ± 0.22	9.91 ± 0.24
*σ*_B_ (Mpa)	24.5 ± 3.4	13.6 ± 1.9	21.4 ± 1.1	24.3 ± 1.8	19.6 ± 0.8	23.1 ± 1.6	25.4 ± 0.6
*ε*_B_ (%)	760 ± 37	561.2 ± 3	516.5 ± 10	468.7 ± 35	633.7 ± 18	536.1 ± 4	493 ± 1

**Table 6:** *J*_c_ values for NR–HSAG and NR–HSAG-SP composites.

Composite n.	1	2	3	4	5	6
**Filler Type**	HSAG	HSAG	HSAG	HSAG-SP	HSAG-SP	HSAG-SP
**Filler Amount (phr)**	5	15	24	5	15	24
***J*_c_ (kJ/m^2^)**	5.2 ± 0.7	3.5 ± 0.3	2.6 ± 0.3	5.4 ± 0.6	4.0 ± 0.4	3.6 ± 0.3
